# AMPK and the Challenge of Treating Hypoxic Pulmonary Hypertension

**DOI:** 10.3390/ijms23116205

**Published:** 2022-06-01

**Authors:** Karen Flores, Patricia Siques, Julio Brito, Silvia M. Arribas

**Affiliations:** 1 Institute of Health Studies, University Arturo Prat, Av. Arturo Prat 2120, Iquique 1110939, Chile; psiques@tie.cl (P.S.); jbritor@tie.cl (J.B.); 2 Institute DECIPHER, German-Chilean Institute for Research on Pulmonary Hypoxia and Its Health Sequelae, 20251 Hamburg, Germany and Iquique 1100000, Chile; 3 Department of Physiology, University Autonoma of Madrid, 28049 Madrid, Spain; silvia.arribas@uam.es

**Keywords:** AMPK, hypoxic pulmonary hypertension, high altitude, cardioprotection

## Abstract

Hypoxic pulmonary hypertension (HPH) is characterized by sustained elevation of pulmonary artery pressure produced by vasoconstriction and hyperproliferative remodeling of the pulmonary artery and subsequent right ventricular hypertrophy (RVH). The search for therapeutic targets for cardiovascular pathophysiology has extended in many directions. However, studies focused on mitigating high-altitude pulmonary hypertension (HAPH) have been rare. Because AMP-activated protein kinase (AMPK) is involved in cardiovascular and metabolic pathology, AMPK is often studied as a potential therapeutic target. AMPK is best characterized as a sensor of cellular energy that can also restore cellular metabolic homeostasis. However, AMPK has been implicated in other pathways with vasculoprotective effects. Notably, cellular metabolic stress increases the intracellular ADP/ATP or AMP/ATP ratio, and AMPK activation restores ATP levels by activating energy-producing catabolic pathways and inhibiting energy-consuming anabolic pathways, such as cell growth and proliferation pathways, promoting cardiovascular protection. Thus, AMPK activation plays an important role in antiproliferative, antihypertrophic and antioxidant pathways in the pulmonary artery in HPH. However, AMPK plays contradictory roles in promoting HPH development. This review describes the main findings related to AMPK participation in HPH and its potential as a therapeutic target. It also extrapolates known AMPK functions to discuss the less-studied HAPH context.

## 1. Introduction

Pulmonary artery hypertension (PAH) is the diagnosis given to patients at rest presenting an increased mean pulmonary arterial pressure (mPAP) due to an increase in pulmonary vascular resistance, vasoconstriction and/or hyperproliferative remodeling of the pulmonary artery, which lead to right ventricular hypertrophy, heart failure and premature death [1,2,3]. In the 6th World Symposium on Pulmonary Hypertension in 2018, the hemodynamic definition of PAH was changed, lowering the mPAP threshold from ≥25 mmHg to >20 mmHg [4]. However, high-altitude pulmonary hypertension (HAPH) is still defined as PAPm ≥ 30 mmHg according to the International Experts Consensus [5]. PAH is classified into five types depending on the cause. Hypoxic pulmonary hypertension (HPH) describes group 3, in which PAH is associated with lung disease and/or hypoxia, chronic obstructive pulmonary disease (COPD), pulmonary fibrosis, obstructive sleep apnea (OSA) and long-term exposure to high altitude [4,6]. In the past three decades, several studies have focused on identifying an ideal therapy for PAH [7], which has been a great challenge, especially for the HAPH subtype, because little is known about it [2]. HAPH is associated with long-term exposure to hypobaric hypoxia, and it is estimated that more than 140 million people worldwide currently live, work or participate in sports at altitudes over 2500 m [3,8]. The general worldwide prevalence of HAPH has been estimated to be between 10% and 15% [5], and the prevalence varies by type of high-altitude exposure. For example, people who permanently live at high altitudes have an HAPH prevalence of 5–18% [5,9,10,11,12], people who visit high-altitude destinations or participate in sports at high altitude have an HAPH prevalence of 4% [13], and those who work at high altitude have an HAPH prevalence of 9% [3]. Based on work-related HAPH observed over the past 20 years, a new type of hypoxia exposure has been described in South America called long-term chronic intermittent hypoxia, and this HAPH subtype has been found in individuals who commute to a work site at high altitude but live at sea level. The rate of hiring people to work under these conditions has increased over time [14]. Therefore, the study of cardiovascular damage due to exposure to high altitude is an important and relevant endeavor.

AMP-activated protein kinase (AMPK) is a heterotrimeric protein kinase consisting of catalytic subunits α and 2 and regulatory subunits β and γ, and it is expressed in various tissues and subcellular locations [15]. AMPK is best known as a sensor of cellular energy status and is involved in restoring energy metabolism homeostasis in cells and whole organs, but AMPK has been implicated in changes to vascular tone and remodeling [16,17,18,19]. Therefore, most researchers have chosen AMPK as the therapeutic target to study cardiovascular diseases, mainly PAH. However, whether AMPK activation mitigates or contributes to the development of HPH continues to be debated. AMPK is activated by drugs, xenobiotics and many physiological factors that increase ATP consumption (heat shock, contraction of skeletal muscle, hypertrophy and cell proliferation) or that decrease ATP production (hypoxia, ischemia and hypoglycemia). These changes lead to an increase in the intracellular ADP/ATP or AMP/ATP ratio, which is detected by AMPK, which then restores the cellular ATP level [20]. Thus, AMPK activates energy-producing catabolic pathways, such as fatty acid and glucose oxidation pathways, and inhibits energy-consuming anabolic pathways of cell growth that deplete energy sources and promote protein synthesis, leading to protection of the cardiovascular system. In HPH, both an increase and decrease in AMPK activation have been observed, and the effect of hypoxia on these changes is still unclear. Studies on the regulation of cardiovascular AMPK activity have reported contradictory conclusions regarding the true effect of AMPK in hypertension; however, most of the literature has focused on AMPK as a potent molecule that mitigates HPH. In recent years, the role of AMPK in HAPH has rarely been studied, and a few HAPH reports have indicated that AMPK activation protects against HAPH. In this review, we describe the main molecular findings related to AMPK participation in HPH pathology and the potential of AMPK as a therapeutic target, and we compare different treatments used to investigate HPH, which has allowed us to understand HAPH despite the lack of information on the function of this kinase in this pathology.

## 2. Structure and Cellular Locations of AMPK

### 2.1. Structure of AMPK

AMPK is a serine/threonine (Ser/Thr) protein kinase that is composed of a heterotrimeric complex comprising α, β and γ subunits; the α subunit has a catalytic function, and subunits β and γ have a regulatory function. Each subunit can be produced in two or more isoforms (α_1_, α_2_, β_1_, β_2_, γ_1_, γ_2_ and γ_3_), which are differentially expressed in various tissues and at different subcellular locations. Each subunit isoform is encoded by multiple genes and can form as many as 12 heterotrimeric AMPK combinations [21,22,23]. All three subunits are required for full AMPK activity. Evidence has shown that heterotrimeric combinations are preferentially activated and play specific roles and that AMP can be regulated by numerous AMPK subunit combinations [21,23,24]. The α subunit contains a canonical N-terminal Ser/Thr kinase domain (KD); an autoinhibitory domain (AID); an adenine nucleotide sensor segment termed an α-linker; and a β subunit-interacting C-terminal domain (α-CTD), which contains an ST loop that harbors the site proposed to be phosphorylated by AKT (also known as protein kinase B (PKB)) [25,26], cAMP-dependent protein kinase (PKA) [27] or glycogen synthase kinase (GSK) [28] and includes the important regulatory threonine 172 (Thr172) residue that is phosphorylated by upstream kinases Ca^2+^/Calmodulin-dependent protein kinase β (CaMKKβ) [29] or liver kinase B1 (LKB1) [30]. The β subunits are composed of a myristoylated, unstructured N-terminus; a carbohydrate-binding module (CBM), sometimes referred to as the glycogen binding domain (GDB); a scaffolding β C-terminal domain (β-CTD) that interacts with both the γ subunit and the α-CTD; and an extended β-linker loop that connects the CBM with the β-CTD. The three alternative γ subunits contain four conserved cystathionine-β-synthase (CBS) domains, which are involved in nucleotide binding (AMP/ADP/ATP) [26,31,32]. The γ subunit of AMPK was first identified by Bateman [33], and it contains four repeats that form two domains. Each of these domains binds one molecule of AMP or an ATP ion in a mutually exclusive manner, consistent with findings showing that high concentrations of ATP antagonize AMPK activation induced by AMP [34].

### 2.2. Localization of AMPK Isoforms in Cardiovascular Tissue

The localization and activation of AMPK follow distinct patterns. The AMPK complex containing the α_2_ subunit is mainly located in tissues with high energy demands (e.g., muscle, brain and liver), and the α_1_ subunit seems to be more widespread and accounts for the majority of the AMPK activity in tissues such as the pancreas and in leucocytes, smooth muscle cells (SMCs) and endothelial cells (ECs), particularly ECs in tissues expressing the α_2_ subunit [24,35]. In the lung, both α_1_ and α_2_ are ubiquitously expressed in pulmonary vessels [36,37,38]. Thus, AMPKα1 is the predominant subunit in ECs and SMCs derived from the pulmonary microvasculature, and AMPKα2 is the predominant subunit in conduit-pulmonary-artery-derived ECs and SMCs [39,40]. In the heart, all AMPK subunit isoforms, except γ_3_, are expressed. The γ_1_ isoform seems to be the major regulatory subunit in all cells; γ_2_ is highly expressed in the heart, and γ_3_ is almost exclusively expressed in fast-twitch skeletal muscle [21,41]. Although the γ_2_ AMPK subunit does not exert the most powerful regulatory effect, it is widely expressed, and the allosteric activation of AMPK complexes containing the γ_2_ subunit is higher than that of those containing the γ_1_ subunit [34]. Mutations in the protein kinase AMP-activated noncatalytic subunit gamma 2 (*PRKAG2*) gene, which encodes the γ_2_ subunit, cause hypertrophic cardiomyopathy [42]; these mutations are exclusively found in nucleotide-binding domains, and some of the resulting mutants are directly involved in binding nucleotides, AMP or ATP [43]. Interestingly, AMPK complexes with different γ subunit isoforms (γ_1_, γ_2_ or γ_3_) display subtle variations in the responses to increases in AMP and ADP, suggesting that AMPK complexes at different locations can be tuned to respond differently to changes in adenine nucleotides, depending on the γ subunit isoform expressed [17,18].

## 3. Function and Regulation of AMPK

AMPK is best known as a sensor of cellular energy status and is involved in the regulation of cellular and whole-organ energy homeostasis [17,18,19,44]. It is activated by energy stress in response to increased ATP consumption (e.g., exercise, cell proliferation and anabolism) or decreased ATP production (e.g., hypoxia, oxidative stress and low glucose levels). Therefore, when the concentration of ATP decreases in a cell, the activation of AMPK is required for restoring ATP levels. Upon activation, AMPK phosphorylates downstream targets to modulate the activities of rate-limiting metabolic enzymes; transcription and translation factors, affecting proliferation and growth pathways either directly or indirectly; and epigenetic regulators. The overall effect of AMPK activation is based on both the cessation of ATP-consuming anabolic pathway activities, such as glucose, protein, cholesterol, triglyceride, fatty acid and ribosomal RNA (rRNA) synthesis, and the promotion of ATP-producing catabolic pathway activities, such as fatty acid and glucose uptake and oxidation and autophagy, to decrease cell growth and proliferation rates [45,46,47,48,49]. The activation of AMPK is dependent on cellular energy status and the activity of upstream stimulatory and inhibitory signaling pathways.

### Regulation of AMPK by Hypoxia

In mammalian cells, various types of metabolic stresses, drugs and xenobiotics activate AMPK through two main mechanisms: the classical or “canonical” activation pathway, which was the first pathway to be described, is triggered by increases in cellular AMP, ADP or Ca^2+^ to activate LKB1 and CaMKKβ, respectively, and the “noncanonical” pathway, which was recently discovered, is triggered by reactive oxygen species (ROS), such as hydrogen peroxide (H_2_O_2_) [50].

The main identified upstream kinases that activate AMPK are LKB1, CaMKKβ and TGF-β-activated kinase 1 (TAK1) [51]. The canonical mechanism explains nucleotide-dependent AMPK activation, i.e., basal AMPK activity is low in cells without stress, but the ATP concentration is decreased under conditions of metabolic stress, and the intracellular ADP/ATP or AMP/ATP ratio is therefore increased; moreover, AMPK has the capacity to detect changes in ATP concentration and reestablish ATP levels [24,30,52,53,54,55]. In cells with a low ATP level, AMPK is activated by three mechanisms:

(I) AMP or ADP binds to the CBS domains of the γ subunit, revealing the Thr172 (human α1 T174) residue in the KD domain of the catalytic α subunit, which is then phosphorylated by LKB1 [17,18,29,56,57,58,59,60].
(II) AMP or ADP binding inhibits Thr172 dephosphorylation by protein phosphatases (PPs) [17,18,30,61,62,63], in contrast to ATP binding, which competitively antagonizes allosteric activation [44,64].
(III) AMP mediates allosteric regulation [17,18,30].

Notably, the degree of allosteric activation depends on the composition of the AMPK complex and, in the case of AMP, is influenced by the concentration of ATP [17,18,59,65].

**Figure 1:**
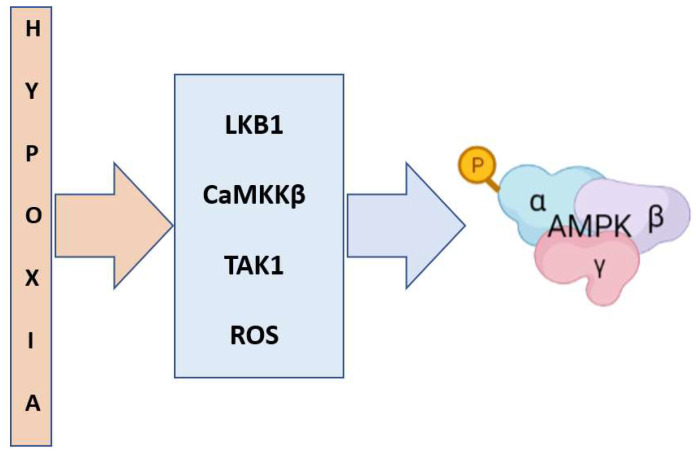
Upstream mediators implicated in AMPK activation by hypoxia.

Hypoxia activates AMPK in various tissues and cell types [66,67]. Specifically, in response to hypoxia, AMPK activity is closely coupled to the inhibition of mitochondrial oxidative phosphorylation through the action of LKB1, the principal upstream kinase that contributes to AMPK activation under metabolic stress conditions [22,29]. The LKB1 complex (comprising LKB1 and accessory subunits STRAD and MO25) [68] appears to be constitutively active under normal conditions [69], and AMPK activation is modulated by adenine nucleotide binding to AMPK [70]. Another canonical mechanism independent of bioenergetic changes involves CaMKKβ, which activates AMPK by phosphorylating Thr172 in the activation loop of the catalytic α-subunit in response to increased cytosolic Ca^2+^ levels [71,72]. The CaMKKβ-AMPK pathway represents an alternate Ca^2+^-activated pathway that induces AMPK activation mediated by hormones that release Ca^2+^ from intracellular stores; these hormones include thrombin [73], ghrelin [74], vascular endothelial growth factor (VEGF) [75], bradykinin [76] and estrogen [77] and are also activated by hypoxia [66,78]. Recent reports have suggested that acute or moderate hypoxia leads to increases in cytosolic calcium, activating AMPK via the upstream kinase CaMKKβ in several cell lines, which operates independently of the AMP/ATP ratio by opening calcium-release-activated calcium (CRAC) channels and inhibiting Na/K-ATPase activity through mitochondrial ROS (mtROS) [66,78]. In addition, ROS are involved in AMPK activation via the noncanonical pathway mediated through an AMP- and LKB1-independent mechanism [79,80,81], suggesting that AMPK is redox-sensitive and functions independent of adenine nucleotides [82]. Studies have shown that mtROS generated as a result of the interaction between nitric oxide (NO) and mitochondrial cytochrome c oxidase activate AMPKα1 in human umbilical vein endothelial cells (HUVECs) under low-oxygen conditions (i.e., 3%) [80,83]. Additionally, H_2_O_2_ has been reported to activate AMPK via oxidative modification of α subunit cysteines; however, the physiological relevance of these modifications has not been fully characterized [64,84]. In addition, TAK1, another kinase capable of direct AMPK activation [85], has been shown to phosphorylate the same site in AMPKα as that phosphorylated by other kinases in an energy-independent manner [86]. TAK1 is a Ser/Thr protein kinase in the mitogen-activated protein kinase (MAP3K) family that plays a crucial role in regulating cell survival, differentiation, apoptosis and inflammatory responses [87]. Both AMPK and TAK1 have been reported to be activated by cytokines such as interleukin-1 (IL-1), tumor necrosis factor (TNF)-α and transforming growth factor-β (TGF-β) [88]. Thus, TAK1 is critical for AMPK phosphorylation under specific conditions, such as inflammation [89]. Figure 1 summarizes the upstream pathways implicated in AMPK activation under hypoxic conditions.

## 4. Hypoxic Pulmonary Hypertension (HPH)

HPH is a common clinical pathophysiological process as well as an important pathological contributor to the development of various heart and lung diseases, such as COPD, chronic pulmonary heart disease and HAPH. Treating PAH is quite challenging, and treating high-altitude-related HPH is even more challenging [90]. However, the treatment strategy for HPH is, in general, similar to that for PAH [91]. HPH is characterized by a progressive increase in pulmonary vascular resistance, pulmonary artery vasoconstriction, perivascular inflammation, hyperproliferative remodeling (including medial hypertrophy with an increase in the number and size of pulmonary artery smooth muscle cells (PASMCs) and intimal proliferation) and PASMC resistance to apoptosis, and these effects increase the pressure of the pulmonary artery and lead to right heart failure and early death [3,92,93,94,95,96,97]. These processes are mainly caused by EC dysfunction, deregulation of the interaction between pulmonary artery endothelial cells (PAECs) and PASMCs, activation of various pathway kinases and ROS production under hypoxic conditions [98,99,100,101]. Hypoxia directly stimulates the endothelium of pulmonary arteries, which results in shear stress modulated by hemoconcentration and increased ROS levels, leading to an imbalance in the expression and secretion of vasoactive molecules and, ultimately, in endothelium impairment and dysfunction [102,103,104]. The consequences include pulmonary vasoconstriction, pulmonary artery remodeling and PAH development [105]. Hypoxic pulmonary vasoconstriction (HPV) is an intrinsic local and adaptive physiological response to alveolar hypoxia that causes constriction of pulmonary arteries to optimize ventilation/perfusion matching, gas exchange and systemic oxygen delivery, as well as to divert blood to better-oxygenated lung segments [106,107,108,109]. The initiation phase of acute HPV is primarily driven by SMC constriction [110]. However, this homeostatic physiological mechanism is damaged under pathological conditions characterized by global and persistent hypoxia, such as lung disease and/or hypoxemia during ascent to altitude, promoting sustained pulmonary vasoconstriction and vascular remodeling, which can cause HPH and right heart failure [2,109,111]. Hypoxia alters the production of endothelial vasoactive mediators such as NO and endothelin-1 (ET-1), induces oxidative stress and decreases EC viability. These changes result in vascular inflammation and damage [112]. Hypoxia-induced vasoconstriction is believed to be a result of released EC-derived contraction factors. The factors identified to date include ROS such as superoxide anions (O_2_^−^), which act by scavenging NO, H_2_O_2_ and endoperoxide; thromboxane A2 (TXA2); and endothelins [113,114]. The scientific consensus indicates that the main factors involved in HPH progression are increased ROS levels, hypoxia-inducible factor (HIF) stabilization and voltage-gated potassium channel Kv1.5 suppression, calcium channel opening and increased intracellular calcium concentration in PASMCs under hypoxia, leading to HPV [115,116,117,118,119]. Previous studies have described the “ROS hypothesis”, suggesting that hypoxia increases mitochondrial ROS generation and elevates the intracellular calcium concentration [120] by inhibiting the activity of Kv channels located in pulmonary arterioles while stabilizing HIF activity. Decreased Kv activity may explain the early pulmonary vascular constriction reaction under hypoxia, and the stabilized HIF axis may activate downstream genes to promote the expression of various hypoxia-related proteins that participate in pulmonary vascular remodeling during HPH through an “ROS/Kv/HIF axis” [119]. ROS affect cell sensitivity to oxidative stress, cell migration, proliferation, apoptosis and matrix protein deposition, all of which are related to vasoconstriction and vascular remodeling [121,122,123]. Thus, HIF-1 contributes to ET-1 expression activation in ECs [124]. In animal models, acute and mild degrees of hypoxia have been shown to cause the rapid expression (within hours) of predominantly vasoconstricting agents such as ET-1, whereas chronic and severe oxygen deprivation stimulates the generation of mitogens such as platelet-derived growth factor-B (PDGF-BB), leading to SMC proliferation and remodeling of the vessel wall. Given that ET-1 is a potent vasoconstrictor that may reduce blood supply to tissue, its increased excretion by ECs into the hypoxic or ischemic environment may be considered representative of EC dysfunction [125]. EC dysfunction deregulates the interaction between PAECs, and PASMCs play crucial roles in the development of PAH [98,99]. Additionally, cytokines/chemokines and growth factors regulate pulmonary endothelial function and influence the development of PAH [126]. Endothelial dysfunction is considered a key underlying feature in most forms of clinical and experimental PAH and is enhanced by inflammatory cytokines/chemokines and growth factors [126,127]. Pulmonary EC dysfunction in PAH patients enhances pulmonary vascular remodeling through an impaired release of vasodilators, such as NO and prostacyclin [128,129,130].

### 4.1. AMPK in HPH

The AMPK molecular pathway involved in HAPH has rarely been studied, and further investigation is needed. Therefore, this section focuses on different AMPK pathways in HPH, comparisons between different hypoxia experiments that induce HPH and the contradictory roles played by AMPK.

AMPK is involved in the response to hypoxia in organ-specific cells such as carotid body type I cells [131], pulmonary arterial SMCs [110] and ECs [132], which monitor O_2_ supply and modulate cardiorespiratory function to maintain arterial partial pressure of oxygen (PaO2) within physiological limits [133].

In recent years, studies have determined the key role played by AMPK in HPH and have proposed it to be a therapeutic target in this pathology. Most of the literature indicates that AMPK plays a vital role in vascular homeostasis, especially under hypoxia, protects against the progression of HPH by activating different signaling pathways and profoundly contributes to cardiovascular protection [15,134,135,136]. However, other authors have indicated that AMPK activation can promote HPH development [51]. In addition, although hypoxia has been shown to activate AMPK in several tissues, at the cardiovascular level in HPH, AMPK activity can be both instigated and inhibited.

#### 4.1.1. AMPK and Pulmonary Artery Vasoconstriction

AMPK plays an important role in HPV due to its high sensitivity to metabolic and oxidative stress under hypoxic conditions [137]. Contradictory mechanisms involve AMPK in HPV, as described by various authors, to promote vasoconstriction or activate vasodilation pathways.

AMPK activation in ECs responds to physiological stimuli, including hypoxia and oxidative stress [138], as well as shear stress [139]. Here, we discuss how AMPK promotes HPH.

With respect to vasodilatation, the main pathway by which AMPK confers protection is related to its antiapoptotic effect on ECs [140] and the activation of endothelial NO synthase (eNOS) upon AMPK phosphorylation at serine 1177, leading to the formation of NO, which is the main vasodilator molecule in the vasculature [141,142,143,144]. At the vascular level, both EC NO production and NO-mediated signaling in SMCs are targets and effectors of the AMPK signaling pathway [142]. Additionally, AMPK exerts a redox-regulatory function by inhibiting the formation of ROS, such as O_2_^−^, through inhibition of the nicotinamide adenine dinucleotide phosphate (NADPH) oxidase complex [145] and an increase in the expression of antioxidant and anti-inflammatory enzymes, such as superoxide dismutase 2 (SOD2), in ECs [146,147]. Nevertheless, many authors have reported a decrease in EC AMPK activation in the pulmonary artery in PAH. Omura et al. [144] found that EC AMPK activity is reduced in distal pulmonary arteries of PAH patients and an experimental mouse model with chronic normobaric hypoxia (10% O_2_)-induced PAH at 4 weeks; specifically, PDGF-BB and fibroblast growth factor-2 (FGF-2) expression in PASMCs was increased, promoting HPH development. This decrease in AMPK activity has been attributed to increased serum levels of inflammatory cytokines, including interferon-γ (IFN-γ) and TNF-α, in PAH patients, demonstrating that inflammatory cytokines impair EC function and phenotype in PAH; however, AMPK activation has been shown to mitigate HPH [144]. Similarly, another study showed that both the activity and expression levels of AMPK were decreased in PAECs in mice with pulmonary hypertension induced by fetal ductus arteriosus constriction; in this case, the decrease in AMPK was attributed to increased expression of protein phosphatase 2A (PP2A) and protein phosphatase 2C (PP2C), and AMPK activation ameliorated PAH [148]. Notably, excessive PP2A activation under pathological conditions results in EC damage or dysfunction by inhibiting AMPK activity [149]. PPs are members of the Ser/Thr protein kinase family and are involved in major intermediary metabolic pathways [150]. At the cardiovascular level, PP2A and PP2C dephosphorylate the Thr172 residue to inhibit AMPK activity [151].

**Figure 2:**
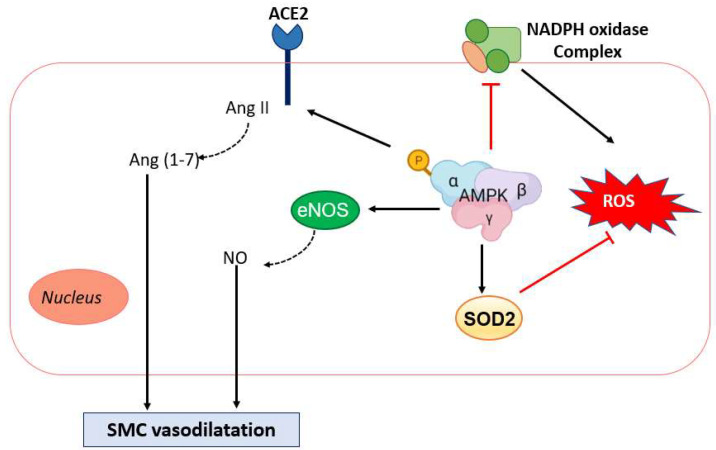
Role of AMPK in PAECs implicated in vasodilatation.

In contrast, some studies have demonstrated that AMPK activation induces vasoconstriction and promotes HPH development [152]. Previous studies have established that hypoxia promotes Ca^2+^-dependent pulmonary artery constriction [115,116,117,118,119]. Notably, both AMPKα1 and AMPKα2 have been described as activated to various degrees depending on the hypoxia level. For example, under mild and severe hypoxia, expression of the AMPKα1 subunit is required to promote HPV, but AMPKα2 subunit expression is only required during severe hypoxia to promote HPV. Thus, it has been shown that AMPKα1 activation induced by LKB1 inhibits Kv1.5 channel currents in PASMCs and leads to pulmonary vasoconstriction in response to moderate and severe hypoxia [117,153]. Evans et al. [39] also postulated that AMPK activation in hypoxia leads to the initiation of Ca^2+^ signaling mechanisms to promote HPV; they proposed that AMPK activation initiates cADPR-dependent Ca^2+^ release from ryanodine-sensitive sarcoplasmic reticulum (SR) stores in PASMCs [39]. Interestingly, Robertson et al. [154] determined that the activation of AMPK is a key event in the initiation of the pulmonary contractile response to acute hypoxia. These findings are supported by some studies that indicate that AMPK is activated in acute hypoxia in the heart and pulmonary artery but not in chronic hypoxia [155,156,157]. These findings indicate that AMPK is essential for the initiation of adaptation to hypoxia at the pulmonary level. The determination of the time of activation of AMPK in acute to chronic hypoxia is still unclear and differs from the methodology used. For example, Viganò et al. [155] observed that acute normobaric hypoxia lasting 48 h at 8% O_2_ in mice causes an increase in AMPK activation, but chronic continuous hypoxia for 10 days at 8% O_2_ does not cause such significant changes. In addition, Kolar et al. [157] showed a decrease in AMPK activation after 21 days of chronic hypoxia. Nevertheless, considering the findings in the literature, the degree and type of hypoxic stress that an individual experiences may also differ, and these differences need to be considered [152]. The finding that AMPKα1 is critical to both Kv1.5 inhibition and HPV is very intriguing. The discovery of single nucleotide polymorphisms (SNPs) in the *PRKAA1* gene (encoding AMPKα1) in native Andean populations that live at and are adapted to high altitude is also interesting [158]. These studies reflect the true roles played by AMPK. Other researchers have shown that the progression from acute to chronic HPH results from excessive repression of AMPK expression in the pulmonary vasculature during sustained chronic hypoxia. Hence, it seems plausible that cardiorespiratory adaptation to hypoxia at altitude and HPH induced by other factors may be driven by cell-specific changes in AMPK subunit expression and/or AMPK activity [159]. Figure 2 depicts the mechanisms implicated in the vasodilatory actions of AMPK.

#### 4.1.2. AMPK and Pulmonary Artery Remodeling

The proliferation and migration of PASMCs are critical processes underlying pulmonary vascular remodeling in HPH [160,161,162]. Therefore, attenuation of PASMC proliferation and pathogenic vascular remodeling is critical for both the prevention and treatment of HPH [163]. PASMCs express both the AMPKα1 and α2 isoforms [164], and various mechanisms are involved in AMPK antiproliferative effects, as confirmed by the activation of AMPK with pharmacological agents. Notably, AMPKα1 has been reported to be involved in decreasing pulmonary artery remodeling by exerting an antiproliferative effect [165,166]. Additionally, Wang et al. [136] showed that knocking out AMPKα2 expression in mice (AMPKα2−/−) exacerbated HPH development. After 4 weeks of exposure to normobaric hypoxia, AMPKα2−/− mice exhibited more severe pulmonary vascular remodeling and PASMC proliferation than did wild-type (WT) mice. In this case, the mTOR/Skp2/p27kip1 signaling axis played a fundamental role. Interestingly, loss of AMPKα2 has been associated with increased phosphorylation of the mammalian target of rapamycin (mTOR), which upregulated S-phase kinase-associated protein 2 (Skp2) and downregulated cyclin-dependent kinase inhibitory protein (p27kip1) expression in PASMCs under hypoxia [136], consistent with previous observations of PASMCs in culture [167]. The mTOR pathway is a major growth-regulating pathway controlled by AMPK. mTOR has been described as a central regulator of protein synthesis; cell growth, proliferation and survival; and autophagy [168]. p27kip1, a cyclin-dependent kinase (CDK) inhibitor, is a critical regulatory protein that exerts an inhibitory effect on mammalian cell proliferation [169], and Skp2 regulates p27kip1 degradation [170]. The inhibitory action of AMPK on the mTOR/Skp2/p27kip1 pathway has been observed with the activation of AMPKα2, which inhibited mTOR activity and downregulated Skp2 expression, preventing p27kip1 degradation and cell proliferation [171]. AMPK can inhibit mTOR activity through phosphorylation and activate tuberous sclerosis complex 2 (TSC2), which together with TSC1 can suppress mTOR activation [172]. Similarly, another study showed that activation of AMPKα2 blocked mTOR phosphorylation in response to PDGF. PDGF activates the PI3K/Akt/mTOR signaling pathway, which in turn upregulates Skp2 and subsequently reduces p27kip1 expression, leading to PASMC proliferation [173,174]. In addition, the PI3K/AKT/mTOR pathway in PASMCs has been shown to be activated through various stimuli, such as ET-1 [175,176], stress and hypoxia [173,177,178]. Additionally, in PAH patients, the mTOR pathway promoted the activation of growth factors such as PDGF, epidermal growth factor (EGF) and FGF, leading to PASMC proliferation [179]. Another interesting molecular pathway in model rats exposed to chronic normobaric hypoxia involved κ-opioid receptor stimulation with U50,488H, a specific κ-opioid receptor agonist, which protected the rats against HPH via AMPK/mTOR pathway activation, inhibiting pulmonary artery remodeling, suppressing PASMC proliferation and inducing PASMC apoptosis [180]. However, in cell culture experiments, both PASMCs and PAECs responded to chronic hypoxia through Akt and mTORC1 activation, which was required for increased proliferation and vascular remodeling [177,181,182]. These results demonstrate the importance of increased AMPKα1/α2 activity, which may indicate a novel therapeutic strategy for the management of HPH.

Another recently proposed AKPK mechanism that may also be a novel therapeutic target in HPH involves the inhibition of ADAM metallopeptidase through thrombospondin type 1 motif 8 (ADAMTS8), a secreted disintegrin that is specifically expressed in the lung and heart. ADAMTS8 expression has been shown to be increased under hypoxia, promoting the proliferation of PASMCs, extracellular matrix (ECM) remodeling and EC dysfunction through autocrine/paracrine signaling. The upregulation of ADAMTS8 expression in PASMCs downregulated AMPK, reduced the apoptosis rate (determined by an increase in the (B-cell lymphoma 2 (Bcl-2)/Bcl-2-associated X protein (Bax)) ratio) and enhanced NOX4-mediated ROS production and PASMC proliferation in patients and animal models within 4 weeks of chronic-hypoxia-induced PAH [183]. Bax promotes cell death, while Bcl-2 prevents apoptosis by inhibiting the activity of Bax [184]. Additionally, NOX4 was upregulated in the PASMCs of mice exposed to chronic normobaric hypoxia, as well as in the lungs of PAH patients [185]. Interestingly, NOX4 has been described as an activator of the mammalian target of rapamycin complex 2 (mTORC2), promoting proliferation and apoptosis-resistant phenotype acquisition by PAH-PASMCs via downregulation of AMPK signaling; in this case, mTORC2 acted as an upstream negative regulator of AMPK signaling, resulting in the activation of mTOR complex 1 (mTORC1) and elevated cell proliferation [186]. Additionally, mTORC1 has been recently described to be a direct inhibitor of AMPK by phosphorylating the α1Ser347/α2Ser345 residues, which is associated with reduced phosphorylation of the Thr172 activation loop. Thus, AMPK and mTOR showed inverse regulatory effects [187]. mTOR is a direct sensor of cellular ATP [188], whereas AMPK is a direct sensor of cellular AMP [57]. Additionally, it has also been described that under severe hypoxia, there is a decrease in cellular ATP, an increase in AMPK activity and inhibition of mTOR activity [189,190]. However, Arsham et al. [191] demonstrated that the hypoxic regulation of the mTOR pathway may be dependent on O_2_ levels and independent of ATP levels, since they observed that mTOR was activated only at low levels of hypoxia, which may subsequently inhibit AMPK activity.

**Figure 3:**
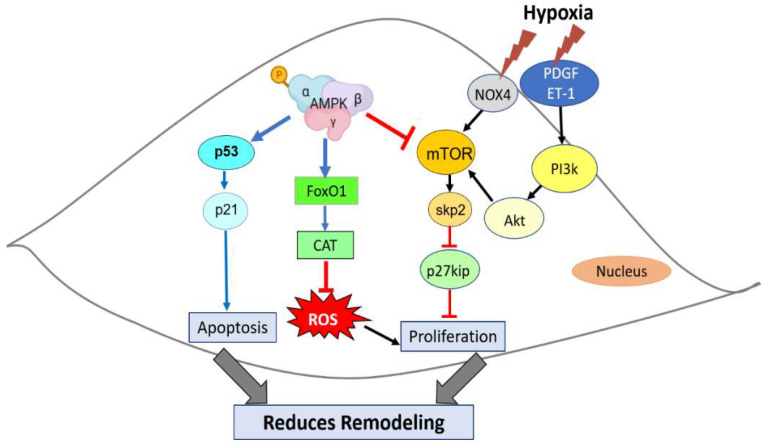
Role of AMPK in reducing PASMC remodeling under hypoxia.

Moreover, AMPK activation or inhibition under hypoxic conditions depends not only on the O_2_ level but also on other factors, such as the redox state of the cell. For example, Awad et al. [192] showed that in a PASMC culture under hypoxia (10% O_2_) for 72 h, increased ROS levels triggered AMPK activation to protect against oxidative stress, which in turn triggered the expression of the transcription factor forkhead box protein O1 (FoxO1) to upregulate catalase (CAT) expression, the major endogenous enzyme scavenger of ROS, including H_2_O_2_. Although this homeostatic mechanism was insufficient to protect PASMCs from hypoxia-induced oxidative stress, the addition of an AMPK activator increased FoxO1/CAT pathway activity, enhancing antioxidant defense. Interestingly, H_2_O_2_ treatment significantly decreased the activation of the AMPK/FoxO1/CAT pathway. Considering these findings, it is clear that ROS production is mediated by hypoxia and that ROS are important in the regulation of survival- and growth-related signaling in SMCs; however, when their production exceeds cellular antioxidant defenses, ROS cause severe damage [193,194] by promoting the progression of pulmonary vascular remodeling in persistent pulmonary hypertension. These data suggested that hypoxic conditions generate an unfavorable cellular environment that leads to excessive ROS production, affecting AMPK activation and reducing its protective effect. In this context, efficient activation of AMPK may trigger a required compensatory mechanism that reestablishes ROS homeostasis and, thus, counteracts HPH progression [192]. Figure 3 depicts the molecular pathways implicated in the inhibitory effect of AMPK on SMC remodeling under hypoxia.

In contrast to other findings regarding the protective effect of AMPK, some researchers postulate that AMPK plays a key role in PAH development by promoting the survival of PASMCs under hypoxic conditions. For example, Ibe et al. [164] demonstrated that mice with chronic-normobaric-hypoxia-induced PAH exhibited increased activation of AMPKα1/α2 in PASMCs and that the addition of the AMPK inhibitor compound C inhibited the activity of both of these isoforms and partially reversed HPH. They observed that the AMPKα1 and AMPKα2 isoforms played differential roles in the survival of PASMCs in HPH. Specifically, activation of AMPKα2 prevented apoptosis, whereas activation of AMPKα1 promoted PASMC survival [164]. In another study, the α-enolase (ENO1) pathway was found to be involved in an AMPK-related mechanism that supports HPH progression; Dai et al. [195] showed that ENO1 levels were elevated in patients with idiopathic PAH (IPAH) and in model mice with normobaric-hypoxia-induced PAH. The overexpression of ENO1 promoted the acquisition of either a proliferative or apoptotic-resistant phenotype in PASMCs via the AMPK-Akt pathway. Because PAH PASMCs exhibit constitutively high AMPK phosphorylation, ENO1 may be critical for maintaining the activation of the AMPK-Akt-GSK3β axis during PAH [195].

## 5. Potential Candidates for HPH Treatment: AMPK as a Therapeutic Target

Over the past 25 years, a large number of investigations into PAH pathology have led to the identification of several effective therapeutic targets, which are mainly found in the endothelin, prostacyclin or NO pathways, and these studies have led to great progress in conventional therapy application and new targeted therapy development. Many of these therapies are based on attenuating the imbalance in the vasoactive mediators that play primary roles in the development and progression of a series of pathological changes in PAH [196,197,198,199]. In this context, AMPK activation has been proposed to be a possible target molecule to reduce pulmonary artery vasoconstriction and vascular remodeling. The true role played by AMPK is still under scrutiny by many researchers trying to develop an effective treatment for HPH; the main findings are described in the following section.

### 5.1. Pharmacological Treatment

Many therapeutic agents used in the treatment of diabetes and atherosclerosis, such as metformin (MET), 5-aminoimidazole-4-carboxamide ribonucleotide (AICAR), thiazolidinediones and statins, have been studied in the PAH context because they exert their vasculoprotective effects through activation of AMPK, potentially conferring protection against PAH [149,200,201]. MET has been assessed and widely used, mostly as a type 2 diabetes drug, for more than 30 years, and AMPK is the central target molecule of MET. The MET mechanism of action involves inhibiting mitochondrial ATP synthesis by inhibiting the activity of complex I in the respiratory chain, thus reducing cellular energy and activating AMPK [202,203]. MET has been demonstrated to activate AMPK in many tissues [204,205,206]. Furthermore, many researchers have shown evidence supporting MET protection against PAH through AMPK activation via different pathways to exert vasodilatory and anti-proliferative effects [174,207,208,209]. The upregulation of AMPK activity in PASMCs induced by MET contributed to decreasing pulmonary vessel remodeling and HPH in rats. Liu et al. [210] presented support for the hypothesis suggesting that MET inhibits HPH in rat models exposed to chronic normobaric hypoxia by inhibiting collagen deposition and proliferation of PASMCs. Another pathway in a nonhypoxic model was found to induce monocrotaline-induced PAH, and AMPK activation induced by MET inhibited pulmonary artery remodeling, leading to a decrease in matrix metalloproteinase-2 (MMP-2) and MMP-9 activity and the expression of tissue inhibitor of metalloproteinase-1 (TIMP-1) [208]. The expression of TIMP-1 in the lungs has been shown to modulate MMP function, which can directly and indirectly regulate the proliferation, migration and apoptosis of ECs and SMCs; hence, MMPs play an important role in the development of PAH [211,212]. The effect of MET via AMPK induction restored angiogenesis and increased the bioavailability of NO, increasing the activity and expression of both eNOS and SOD2 and disrupting the eNOS-caveolin-1 association in ECs [148]. These results indicate that EC AMPK plays protective roles against hypoxia-induced PAH and would be a novel therapeutic target for the treatment of HPH [144].

Another pharmacological agent, AICAR, is an AMP analog and is widely used to activate AMPK in experiments. Studies have indicated that AMPK activation by AICAR significantly attenuates HPH in mice. AICAR has been observed to reduce mPAP, PASMC proliferation and the degree of vascular remodeling in lungs via increased protein expression and phosphorylation of AMPKα1 in rats exposed to 8 hr of chronic intermittent normobaric hypoxia per day for 4 weeks. In this case, although hypoxia increased the activation of AMPK, AICAR was required to sufficiently enhance its activation to reduce vascular remodeling [165]. In a similar experiment, rats with PAH exposed to 8 hr of chronic intermittent normobaric hypoxia per day were treated with the pharmacological agents salidroside and AICAR, which confers protection against HPH by inducing AMPKα1 activation in PASMCs; in summary, these agents may have reduced cell proliferation by affecting the P53-P21/P27-PCNA pathway and may have enhanced cell apoptosis by affecting the P53-Bax/Bcl-2-caspase 9-caspase 3 pathway [166]. AMPK plays an important role in the regulation of p53 and p21, as observed by Zhuang et al. [213], who also found that AMPK-p53-p21 pathway activation was downregulated in the lungs and pulmonary arteries of rats with monocrotaline-induced PAH. Specifically, this group showed that the activation of AMPK increased the expression of p53 and p21 and inhibited PASMC proliferation that had been induced by PDGF-BB [213]. Another mechanism mediating the beneficial effects of AMPK on HPH involves AMPK-ACE2 axis activation. Specifically, AMPK activated by AICAR phosphorylates angiotensin-converting enzyme 2 (ACE2) at Ser680 in ECs and inhibits the murine double minute 2 (MDM2)-mediated ubiquitination of ACE2, thereby mitigating pulmonary hypertension in patients with idiopathic PAH and mice with HPH by increasing vasodilation [214,215,216]. Additionally, ACE2 stability was increased under conditions in which AMPK was activated by MET treatment [202,217]. MDM2 is an E3 ubiquitin ligase with increased expression in patients and animal models of PAH, and it ubiquitinates ACE2, contributing to PAH development [215]. Statins may also be a promising therapeutic treatment for HPH related to their anti-inflammatory action and metabolic regulatory effects mediated via AMPK [218]. In addition, several small molecules that directly activate AMPK have been identified, such as A769662, 991 and MT 63-78, and have been shown to ameliorate HPH [59,219,220].

### 5.2. Phytochemical Treatment

Some phytochemicals are candidates for possible PAH prevention. For example, resveratrol (RSV), a polyphenolic compound found at high concentrations in grapes and red wine, has antihypertensive, antioxidant and anti-inflammatory properties and can upregulate eNOS expression and scavenge OH/O_2_^−^ and peroxyl radicals, which can inhibit lipid peroxidation [221,222]. Thus, RSV may reverse pulmonary vasculature remodeling and alleviate the HPH severity induced by chronic hypobaric and normobaric hypoxia [162,223]. Interestingly, the activation of AMPK by RSV inhibited SMC contractility by inhibiting Ang-II-induced phosphorylation of myosin phosphatase-targeting subunit 1 (MYPT1) and myosin light chain [224]. At the endothelial level, RSV has been shown to increase NO production and promote vasodilation through activation of the LKB1/AMPK/eNOS signaling axis [221,225,226,227]. Berberine is another phytocomposite found in plants from the family Berberis with anti-inflammatory and antioxidant activities, including beneficial vascular effects in hypertension. For example, berberine reduced endothelium-dependent contractions, probably by activating AMPK, thus inhibiting endoplasmic reticulum stress and subsequently promoting ROS scavenging and leading to downregulated cyclooxygenase-2 (COX-2) expression [228].

### 5.3. Adipokine Treatment

**Figure 4:**
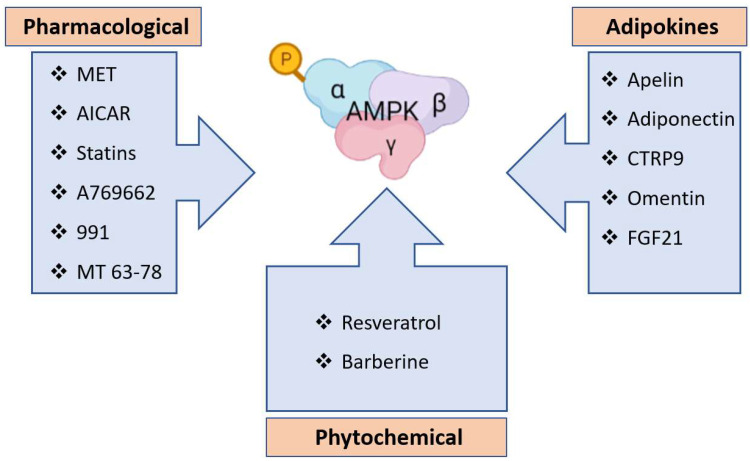
Summary of candidates for HPH treatment through AMPK activation.

Adipokines are derived from adipose tissue [229,230]. The effects of certain adipokines on the activation of AMPK in the vascular system and PAH mitigation have been studied. For example, apelin was found to be a potent regulator of vascular function. Notably, exogenous apelin administration exerted a vasodilatory effect via eNOS pathway activation [231,232,233,234]. Apelin has been recently described as a ligand for the G-protein–coupled receptor APJ (APLNR) [235], and both apelin and APLNR are highly expressed in the lungs, especially in the endothelium of the pulmonary vasculature [236]. Chandra et al. [237] demonstrated that mice lacking the Apelin gene developed worsened PAH in response to hypoxia and that this outcome was mediated by downregulation of eNOS expression. Previous studies presented in this review indicated that this effect was caused by decreased AMPK activation, which may have led to both decreased expression of KLF2 and reduced eNOS phosphorylation, suggesting that AMPK is a critical intermediary mediator of Apelin-APJ signaling in PAECs. In addition, patients with PH were found to have significantly reduced levels of serum apelin, suggesting that disruption of apelin signaling contributes to the pathogenesis of the clinical disease [237]. Adiponectin, another adipokine thought to prevent PAH through AMPK activation, is secreted in large quantities from adipose tissue. Upon binding to its receptors AdipoR1 and R2, adiponectin initiates a series of tissue-dependent signal-transduction-triggered processes, including AMPK phosphorylation [238]. Adiponectin has been considered a potent biomarker of PAH [239]. Adiponectin exerts pleiotropic effects on inflammation and cell proliferation and, thus, plays a potential role in maintaining pulmonary vasculature integrity [179]. Nakagawa et al. [240] reported that the intravenous administration of adenovirus harboring full-length mouse adiponectin in mice exposed to chronic normobaric hypoxia (10%) led to ectopic adiponectin expression, which significantly suppressed pulmonary arterial wall thickening and right ventricular hypertrophy (RVH). The adiponectin/AMPK activation pathway would be a potential therapeutic target in PAH. Lou et al. [241] developed a treatment that involved a combination of adipose-derived stem cells (ADSCs) with adiponectin. Specifically, the transplantation of ADSCs containing adiponectin suppressed PASMC proliferation in PAH rats by activating the AMPK/BMP/Smad pathway. The BMP/Smad signaling pathway is downstream of AMPK signaling in the adiponectin regulatory pathway, which plays a crucial role in the antiproliferation of PASMCs [241]. Another adipokine with vasculoprotective effects is C1q/TNF-related protein-9 (CTRP9). CTRP9 is a member of the adipokine family and has been identified as an adiponectin paralog [242,243] involved in lipid metabolism [244] and cardiovascular protection [245,246]. The vasorelaxative adipocytokine CTRP9 promoted endothelium vasorelaxation mediated via the AdipoR1/AMPK/eNOS/NO signaling pathway [243] to protect against endothelial impairment and vascular remodeling [247]. Interestingly, in a rat model of HPH induced by exposure to chronic intermittent hypobaric hypoxia (8 h/day), a dose-dependent decrease in the serum concentration of CTRP9 was observed, and the overexpression of CTRP9 in lung tissues was induced by an adeno-associated virus (AAV-CTRP9) vector that mitigated HPH by reducing ET-1 production and inactivating ERK1/2 in pulmonary ECs [91]. Another adipokine, omentin, has been observed to exert an anti-inflammatory effect on vascular ECs to prevent TNF-α-induced COX-2 expression by inhibiting JNK activation, presumably through the activation of the AMPK/eNOS/NO pathway [248]. It was recently observed that omentin may confer protection against hypertension development by inhibiting vascular structural remodeling and inhibiting PDGF-BB-induced vascular SMC migration by mediating an antioxidative mechanism [249]. Another factor that exerts protective effects in HPH is fibroblast growth factor 21 (FGF21), which has the beneficial effect of protecting blood vessels. FGF21 is a member of the fibroblast growth factor family and is an endocrine factor secreted primarily by the liver. FGF21 is expressed in the AMPK/PGC-1α pathway and promotes peroxisome proliferator-activated receptor γ (PPARγ) expression, a ligand-activated nuclear transcription factor, and in HPH model mice exposed to chronic intermittent normobaric hypoxia (10%) for 8 h/day, FGF21 effectively inhibited PH [250]. Figure 4 summarizes the main candidates for HPH treatment through AMPK activation.

## 6. Conclusions

This review aimed to provide a better understanding of the role of AMPK functions in HPH. Despite some controversial findings, the majority of available data indicate that AMPK plays a key role in antiproliferative, antihypertrophic and antioxidant pathways in the pulmonary vasculature and support the notion that its activation may be a potential therapeutic target in the treatment of HPH. The review also provides information that may be useful to explain the role of AMPK in the HAPH context, which has been insufficiently studied to date.

### Limitation

Little information is available to explain the role of AMPK in HAPH.

## Data Availability

Not applicable.

## Abbreviations

ACE2	angiotensin-converting enzyme 2
ADAMTS8	ADAM metallopeptidase with thrombospondin type 1 motif 8
ADSC	adipose-derived stem cells
AICAR	5-aminoimidazole-4-carboxamide ribonucleotide
AID	autoinhibitory domain
AKT	protein kinase B
AMPK	AMP-activated protein kinase
Ang II	angiotensin II
Angiotensin (1-7)	Ang (1-7)
APLNR	G-protein–coupled receptor APJ
Bax	Bcl-2-associated X protein
Bcl-2	B-cell lymphoma 2
CBM	carbohydrate-binding module
CBS	cystathionine-β-synthase
CaMKKβ	Ca^2+^/Calmodulin-dependent protein kinase β
CAT	catalase
COPD	chronic obstructive pulmonary disease
COX-2	cyclooxygenase-2
CRAC	calcium-release-activated calcium channel
α-CTD	α C-terminal domain
CTRP9	C1q/TNF-related protein 9
ECs	endothelial cells
ECM	extracellular matrix
EGF	epidermal growth factor
ENO1	α-enolase
eNOS	endothelial NO synthase
ET-1	endothelin-1
FGF-2	fibroblast growth factor-2
FoxO1	forkhead box protein O1
GDB	glycogen-binding domain
GSK	glycogen synthase kinase
HAPH	high-altitude pulmonary hypertension
HIF	Hypoxia-inducible factors
HPH	hypoxic pulmonary hypertension
HPV	hypoxic pulmonary vasoconstriction
HUVECs	human umbilical vein endothelial cells
H_2_O_2_	hydrogen peroxide
IFN-γ	interferon-γ
IL-1	interleukin-1
KD	kinase domain
LKB1	liver kinase B1
MAP3K	mitogen-activated protein kinase kinase kinase
MDM2	murine double minute 2
MET	metformin
MMP-2/9	matrix metalloproteinase-2/9
MYPT1	myosin phosphatase-targeting subunit 1
mtROS	mitochondrial ROS
mTOR	mammalian target of rapamycin
mTORC2	mammalian target of rapamycin complex 2
mTORC1	mammalian target of rapamycin complex 1
NADPH oxidase	nicotinamide adenine dinucleotide phosphate oxidase
NO	nitric oxide
OSA	obstructive sleep apnea
O_2_	oxygen
O_2_^−^	superoxide anions
PAPm	mean pulmonary arterial pressure
PAECs	pulmonary artery endothelial cells
PAH	pulmonary artery hypertension
PASMCs	pulmonary artery smooth muscle cells
PaO2	arterial partial pressure of oxygen
PDGF-BB	platelet-derived growth factor-BB
PKA	cAMP-dependent protein kinase
PPs	protein phosphatases
PP2A	protein phosphatase 2A
PP2C	protein phosphatase 2C
PRKAG2	protein kinase AMP-activated noncatalytic subunit gamma 2
p27kip1	cyclin-dependent kinase inhibitory protein
rRNAs	ribosomal RNAs
ROS	reactive oxygen species
RSV	resveratrol
RVH	right ventricular hypertrophy
Skp2	S-phase kinase-associated protein 2
SMCs	smooth muscle cells
SNPs	single nucleotide polymorphisms
SOD2	superoxide dismutase 2
SR	sarcoplasmic reticulum
TAK1	TGF-β-activated kinase 1
TGF-β	transforming growth factor-β
TIMP-1	tissue inhibitor of metalloproteinase-1
TNF-α	tumor necrosis factor-α
TSC1	tuberous sclerosis complex 1
TXA2	thromboxane A2
TSC2	tuberous sclerosis complex 2
VEGF	vascular endothelial growth factor
